# Social Isolation and Cognitive Aging: Are Operational Definitions Creating a Reproducibility Crisis?

**DOI:** 10.1093/geroni/igaf122.1859

**Published:** 2025-12-31

**Authors:** Ruijia Chen, Dylan Tran, Jingxuan Wang, Erin Ferguson, Mary Thoma, Ashwin Kotal, Jacqueline Torres, M Maria Glymour

**Affiliations:** Boston University School of Public Health, Boston, Massachusetts, United States; Boston University, Boston, Massachusetts, United States; University of California, San Francisco, San Francisco, California, United States; University of California, San Francisco, San Francisco, California, United States; University of California, San Francisco, San Francisco, California, United States; University of California, San Francisco, San Francisco, California, United States; University of California, San Francisco, San Francisco, California, United States; Boston University, Boston, Massachusetts, United States

## Abstract

Research on social isolation and health shows conflicting findings, potentially due to methodological differences in defining social isolation. We conducted multiverse analyses to examine how varying definitions affect associations with cognitive function and decline. Using the 2010-2020 Health and Retirement Study (n = 12,975), we defined social isolation using 16 items related to marital status, living arrangements, social interaction frequency, and participation in social activities. We created continuous and binary composite scores based on all possible combinations of these items, using top tertile and quartile cutoffs for binary definitions. Cognitive function was measured using the Telephone Interview for Cognitive Status, with scores standardized to baseline. Linear mixed-effects models evaluated associations between each definition and cognitive level/decline, adjusting for age, sex, race, and education. Across 196,587 model choices, most models indicated a negative association between social isolation and cognitive function level, with point estimates ranging from very large to nearly null (-0.31 to 0.01 standard deviations average difference across binary social isolation specifications). Most models also suggested social isolation was associated with faster cognitive decline (-0.001 to 0.03 standard deviations average annual difference across binary specifications). While social isolation appears associated with lower cognitive function levels and faster cognitive decline, the magnitude of these associations varies widely depending on how social isolation is defined.

